# Expression of FBXW11 in normal and disease‐associated osteogenic cells

**DOI:** 10.1111/jcmm.17767

**Published:** 2023-05-17

**Authors:** Luca Dalle Carbonare, Macarena Gomez Lira, Arianna Minoia, Jessica Bertacco, Silvia Orsi, Angela Lauriola, Veronica Li Vigni, Alberto Gandini, Franco Antoniazzi, Donato Zipeto, Monica Mottes, Lekhana Bhandary, Daniele Guardavaccaro, Maria Teresa Valenti

**Affiliations:** ^1^ Department of Medicine University of Verona Verona Italy; ^2^ Department of Neurosciences, Biomedicine and Movement Sciences University of Verona Verona Italy; ^3^ Department of Biotechnology University of Verona Verona Italy; ^4^ Department of Surgery, Dentistry, Pediatrics and Gynecology University of Verona Verona Italy; ^5^ Flaskworks, LLC Boston Massachusetts USA

**Keywords:** cleidocranial dysplasia, differentiation, FBXW11, mesenchymal stem cells, osteosarcoma

## Abstract

The ubiquitin‐proteasome system (UPS) plays an important role in maintaining cellular homeostasis by degrading a multitude of key regulatory proteins. FBXW11, also known as b‐TrCP2, belongs to the F‐box family, which targets the proteins to be degraded by UPS. Transcription factors or proteins associated with cell cycle can be modulated by FBXW11, which may stimulate or inhibit cellular proliferation. Although FBXW11 has been investigated in embryogenesis and cancer, its expression has not been evaluated in osteogenic cells. With the aim to explore FBXW11gene expression modulation in the osteogenic lineage we performed molecular investigations in mesenchymal stem cells (MSCs) and osteogenic cells in normal and pathological conditions. In vitro experiments as well as ex vivo investigations have been performed. In particular, we explored the FBXW11 expression in normal osteogenic cells as well as in cells of cleidocranial dysplasia (CCD) patients or osteosarcoma cells. Our data showed that FBXW11 expression is modulated during osteogenesis and overexpressed in circulating MSCs and in osteogenically stimulated cells of CCD patients. In addition, FBXW11 is post‐transcriptionally regulated in osteosarcoma cells leading to increased levels of beta‐catenin. In conclusion, our findings show the modulation of FBXW11 in osteogenic lineage and its dysregulation in impaired osteogenic cells.

## 1 BACKGROUND

An important role in post‐translational protein modification is performed by the ubiquitin‐proteasome system (UPS) which regulates protein degradation.^1^ Removal of defective or redundant proteins is important for maintaining cellular homeostasis and for regulating signal transduction. The presence of damaged material or proteins that are not functional for cellular homeostasis would cause serious damage and cellular alterations.^2^ Importantly, microRNAs post‐transcriptionally regulate the ubiquitin‐proteasome system and it has been demonstrated that miR 221 targeting E3 ubiquitin‐protein ligase homologue (MDM2) is able to modulate p53 (TP53) in hepatocellular carcinoma.^3^ The identification of proteins bound to degradation is granted to proteins belonging to the F‐box family, involved in many processes including haematopoietic regulation and neoplastic transformation.^4^ Based on the protein structure, the F‐box family is divided into the subfamilies FBXW (F‐box protein with WD40 domains), FBXL (F‐box protein with leucine‐rich repeats) and FBXO (F‐box protein with other domains), each of which can identify specific target proteins for ubiquitination.^5^, ^6^ The FBXW subfamily is composed by 10 members, such as FBXW1, 2, 4, 5 and 7–12. In particular, FBXW1 is also named b‐TrCP1 and FBXW11 is also known as b‐TrCP2.^7^ These two members (b‐TrCP1 and b‐TrCP2) have been well characterized and are involved in cell cycle progression and migration as well as in the regulation of signal transduction.^7^ b‐TrCP1 is encoded by the BTRC gene on chromosome 10, while b‐TrCP2 (also known as FBXW11, FBXW1B and HOS) is encoded by the FBXW11 gene on chromosome 5.^7^ It has been suggested that b‐TrCP1 and b‐TrCP2 are redundant proteins with similar features. However, it has been reported that silencing b‐TrCP2 reduces cell growth, while b‐TrCP1 silencing shows the opposite effect and promotes cell growth. In addition, silencing b‐TrCP2 reduces cell migration and induces autophagy while silencing b‐TrCP1 does not produce such effects.^8^ These findings suggest that b‐TrCP1 and b‐TrCP2 have no redundant functions and that b‐TrCP2 is mostly involved in the control of cellular processes.

FBXW11 can modulate transcription factors and regulators associated with the cellular cycle. As FBXW11 can have stimulatory or inhibitory effects on cellular proliferation, a cell type‐dependent role has been suggested for it.^5^ These opposed effects are a consequence of cell cycle activator or suppressor substrates, both of which are potential targets of FBXW11.

In colorectal cancer, FBXW11 promotes stem‐cell‐like features and migration thereby contributing to tumorigenesis.^9^ On the contrary, FBXW11 suppression promotes tumour proliferation in non‐small cell lung cancer cells.^10^ Wang et al.^11^ observed lower expression of FBXW11 in long‐term haematopoietic stem cells whereas the expression of FBXW11 was higher in short‐term haematopoietic stem cells and in various haematopoietic progenitor cells, thus suggesting that FBXW11 is mostly expressed in mature progenitor cells. Overexpression of FBXW11 has been observed in acute lymphocytic leukaemia and it plays an important role in haematopoietic stem cells repopulation.^5^, ^12^ Furthermore, FBXW11 can be post‐transcriptionally regulated by microRNAs. In studies performed on tumour cells it has been demonstrated that FBXW11 is a target of miR 221.^13^ In osteosarcoma, it has been reported that miR 221 promotes cisplatin resistance.^14^

Although FBXW11 has been studied in haematopoietic stem cells and in various types of tumours, no studies have ever been performed to evaluate the role of FBXW11 in the osteogenic lineage.

It is known that the osteogenic lineage derives from mesenchymal stem cells (MSCs) whose commitment is induced by the transcription factor RUNX2. RUNX2 is considered the master gene of osteogenesis and its expression is regulated by different cellular signals.^15^ In particular, the WNT/β‐catenin pathway induces RUNX2 expression during osteogenic commitment.^16^ RUNX2 can also be regulated post‐transcriptionally and we recently demonstrated that RUNX2 protein levels are increased in miR‐204‐silenced MSCs during the middle phase of osteogenic differentiation.^17^ Moreover, using melanoma cells KO for the RUNT domain of RUNX2, we demonstrated that the RUNT domain plays an important role in the modulation of the expression of genes involved in bone metastases.^18^

The present work, therefore, contributes to the understanding of FBXW11 role in osteogenic cells by evaluating its modulation both during osteogenic commitment and in altered osteoblastic cells characterized by RUNX2 mutations or resulting from tumour transformation.

## 2 MATERIALS AND METHODS

### 2.1 Cells

Human mesenchymal stem cells (hMSCs) obtained from human bone marrow where purchased from PromoCell (C‐12974; PromoCell). PromoCell reported that these hMSCs where tested for cellular morphology, proliferation ability, viability and they are characterized by flow cytometric analysis of a comprehensive panel of markers, namely CD73/CD90/CD105 and CD14/ CD19/CD34/CD45/HLA‐DR as proposed by the ISCT (https://promocell.com/wp‐content/uploads/product‐information/manual/C‐12974.pdf). For the experiments of this study we used hMSCs at three passage. hMSCs were plated at a density of 5 × 10^4^ cells in presence of mesenchymal stem cell growth medium (PromoCell) or osteogenic differentiation medium (PromoCell). Cells were incubated at 37°C with 5% CO_2_.

Alkaline phosphatase staining was performed in MSCs after 3, 7 and 14 days of differentiation in 96 well‐plate and fixed with 4% paraformaldehyde (P6148; Sigma‐Aldrich) by using the Alkaline Phosphatase staining kit (SCR004; Merck Millipore). The stained area has been calculated using Image J as previously reported.^19^

Human osteosarcoma cell lines (MG63‐ CRL‐1427aliquots used at five passage) and U2OS‐ HTB‐96 (aliquots used at, four passage) were purchased by ATCC and cultured with high‐glucose DMEM (ECB7501L; EuroClone) as previously reported.^20^ Primary human osteoblasts (HOB, aliquots used at four passage) were purchased by Promocell (C‐12720) and cultured in presence of osteoblast growth medium (C‐27001; PromoCell).

To evaluate RUNX2 degradation in RUNX2 or RUNT KO melanoma cells we used A375 (obtained by from American Type Culture Collection) or A375‐3G8 melanoma cell line, respectively. In particular, the RUNT‐KO A375‐3G8 cell line was generated in our laboratory by CRISPR/Cas9 from parental A375 cells as we previously reported.^21^ Both A375 or A375‐3G8 melanoma cells were cultured in presence of DMEM/F12 containing 10% FBS (foetal bovine serum) supplemented with antibiotics (1% penicillin and streptomycin) and 2 mM of glutamine. The proteasome or proteome inhibition was performed by using MG132 (50 μM; Calbiochem) or cycloheximide (CHX 100 μg/mL; Sigma Aldrich) as previously reported.^22^

### 2.2 Patients

Skin biopsies and peripheral blood were obtained from two cleidocranial dysplasia (CCD) paediatric patients ([P1: mutation: c.897 T > G‐ > p (Tyr299*); 8‐year‐old male] and [P2: c.1019del‐ > p (Ser340*);10‐year‐old female]) and from age‐matched healthy donors.

Parents of each patient and donor gave informed consent. The analyses performed in patients or donors samples were approved by the ethical committee of Azienda Ospedaliera Universitaria Integrata of Verona, Italy (number 1538; 3 December 2012; local ethical committee of Azienda Ospedaliera Integrata di Verona).

Human dermal fibroblast cultures were obtained from explanted skin biopsies as we previously reported.^23^ The cells (used at five [P1], four [P2] and five [P2] passage) were cultured with high‐glucose DMEM (ECB7501L; EuroClone) in presence of 10% FBS (10270–106, Gibco; Life Technologies Limited), 2 mM l‐glutamine (5‐10 K00‐H; BioConcept AG), 100 U/mL penicillin and 100 μg/mL streptomycin (penicillin–streptomycin; ECB3001D; EuroClone), as previously described.^23^

Circulating mesenchymal stem cells (cMSCs) were obtained from peripheral blood as we previously described.^24^ In particular, cMSCs were isolated from peripheral blood using two Ficoll procedures. Then, 200 μL of an antibodies cocktail (RosetteSep Mesenchymal Enrichment Cocktail; code #15128; StemCells) were applied to concentrated mononuclear cells mixed with 4 mL of additional peripheral blood. The antibodies (to glycophorin A, CD3, CD14, CD19, CD38 and CD66b [Stem Cell Technologies, Inc.]) cross linked the unwanted cells. Analysis of cMSCs phenotype has been performed as previously reported.^23^ Briefly, the expression of CD3, CD14, CD19, CD45 and CD34 haematopoietic markers as well as MSC‐positive markers CD73 and CD105 at RNA levels has been performed. This method is used to perform phenotypic analyses for cells obtained with stringent stem cell purification techniques.^25^

### 2.3 Cell transfections

MSCs were plated into T25 flasks and differentiated to the osteogenic lineage. At cell confluency of 60%–70% of confluence, transfection was performed using Lipofectamine 3000 Reagent (L3000‐008; Invitrogen by Thermo Fisher Scientific Baltics UAB) as previously reported.^17^ To force the expression of RUNX2, cells were transfected with Silencer Select Pre‐designed siRNA anti‐miR‐204‐5p (Cat#: AM17000, ID: AM11116; Ambion by Thermo Fisher Scientific) as previously reported.^17^ To dowregulate FBXW11 expression, cells were transfected with Silencer Select Pre‐designed siRNA anti‐FBXW11 (Cat#4392420, ID: s23487; Ambion by Thermo Fisher Scientific) and scramble‐negative control (Cat#: AM17010; Ambion by Thermo Fisher Scientific). We used the following concentrations as indicated by manufacturer's instructions: siRNA anti‐miR‐204‐5p, 197 pmol/T25 flask; siRNA anti‐FBXW11 591 pmol/T75 flask. The effects of silencing was evaluated by gene expression or WB analyses.

After 48 h of transfection, cells were collected. Cell pellets were processed for RNA or protein extraction as previously reported.^17^

### 2.4 RNA extraction and reverse transcription

Pellets were collected and stored at −80°C until use. Then, total RNA was extracted by using an ‘RNeasy® protect mini kit’ (Qiagen), as we previously reported.^26^ MicroRNAs were extracted by using the miRNeasy Quiagen mini kits (Qiagen). ‘Qubit™ RNA HS assay kit’ (Invitrogen) and a Qubit 3 Fluorometer (Invitrogen by Thermo Fisher Scientific; REF Q3321) was used to evaluate the quantity and quality of RNA samples. Total RNA was reverse‐transcribed with the first‐strand cDNA synthesis kit (GE Healthcare). microRNAs were transcribed by using TaqMan MicroRNA Reverse Transcription Kit (Thermo Fisher Corporation; 4366596). Then, cDNA samples were maintained at −80°C until use.

### 2.5 Real‐time PCR

Gene expression was analysed by performing real‐time PCR as reported previously.^24^ Predesigned, gene‐specific primers and probe sets for each gene (RUNX2, Hs1047973_m1; FBXW11 Hs00606870_m1; SP7 Hs00541729_m‐1; SPP1 hs00167093_m1; ALPL Hs01029144_m1; BGLAP Hs01587814_g1; 001973; B2M, hs999999_m1 [housekeeping]; GAPDH, 0802021 [housekeeping]) were obtained from assay‐on‐demand gene expression products (Thermo Fisher Corporation). To evaluate the post‐transcriptional modulation of FBXW11 by microRNA in osteosarcoma cells, we evaluated the expression of miR 221 by using miR‐221‐3p, 000524 and U6 snRNA (Thermo Fisher Corporation). TaqMan SDS analysis software (Applied Biosystems) was used to identify Ct values as reported previously.^24^ Relative gene expression levels were calculated by using the 2^−ΔΔCT^ method as previously reported.^24^

### 2.6 Digital droplet PCR

To analyse the expression of RUNX2 and FBXW11 in cMSCs, we used a digital droplet PCR (ddPCR) test as previously reported.^23^ In particular, five microliters of cDNA at a concentration of 0.2 ng/μL were added to ddPCR supermix for no UTP probes (10 μL), with 1 μL of RUNX2 or FBXW11 (FAM‐MGB) and B2M (VIC‐NGB) control TaqMan probe (Applied Biosystems). The mix was added to the QX200 droplet generator (BioRad) containing 70 μL of oil. Droplets were carried out to a 96‐well plate and then heat‐sealed with a tinfoil sheet.

The thermocycling conditions were: Step 1: pre‐incubation, 95°C for 10 min, Step 2: amplification, 95°C for 30 s, Step 3: annealing, 60°C for 1 min, Step 4: heat inactivation, 98°C for 10 min; Step 2 and 3 were repeated for 40 cycles. Plates with droplets were placed in a reader for QX200 droplet. The droplets were analysed individually, through a two‐colour detection system (FAM and VIC). QuantaSoft (1.7.4.0917 © 2021; Bio‐Rad) was used to process the results according to the manufacturer's instructions.

### 2.7 Western blotting

Proteins were separated by using SDS‐PAGE. Western blot analyses were performed as previously reported.^27^ Briefly, proteins were extracted with the RIPA buffer (Thermo Fisher Scientific). BCA assay (Thermo Fisher Scientific) was used to quantify the proteins. The proteins were separated by using sodium dodecyl sulphate‐polyacrylamide gel electrophoresis (SDS‐PAGE). Then, the proteins were transferred onto polyvinylidene difluoride membranes (Thermo Fisher Scientific) and probed with primary antibodies (RUNX2; Cell Signalling; 8486) (FBXW11; PA5‐109715; Invitrogen) (BetaCatenin; PA5‐19469; Invitrogen) (β‐actin; BA3R) (Thermo Scientific) and secondary antibodies anti‐mouse (Cell Signalling; 7076) and anti‐rabbit (Cell Signalling; 7074). Signals were detected with a method through the chemiluminescence reagent (ECL; Millipore), as previously reported.^27^ LAS4000 Digital Image Scanning System (GE Healthcare) was used to acquire the images and densitometric analyses were performed by ImageQuant software (GE Healthcare) as previously reported.^27^

### 2.8 Statistical analysis

The Results were expressed as mean ± SD. The statistical analysis was performed by using the Mann–Whitney test (to compare two groups) or one‐way anova (to compare more than two groups). Analyses were applied to experiments performed six times; the outliners have been enclosed in the analyses. For the data analyses, SPSS for Windows, version 22.0 (SPSS Inc.) was used.

## 3 RESULTS

### 3.1 FBXW11 expression during osteogenesis

**FIGURE 1:**
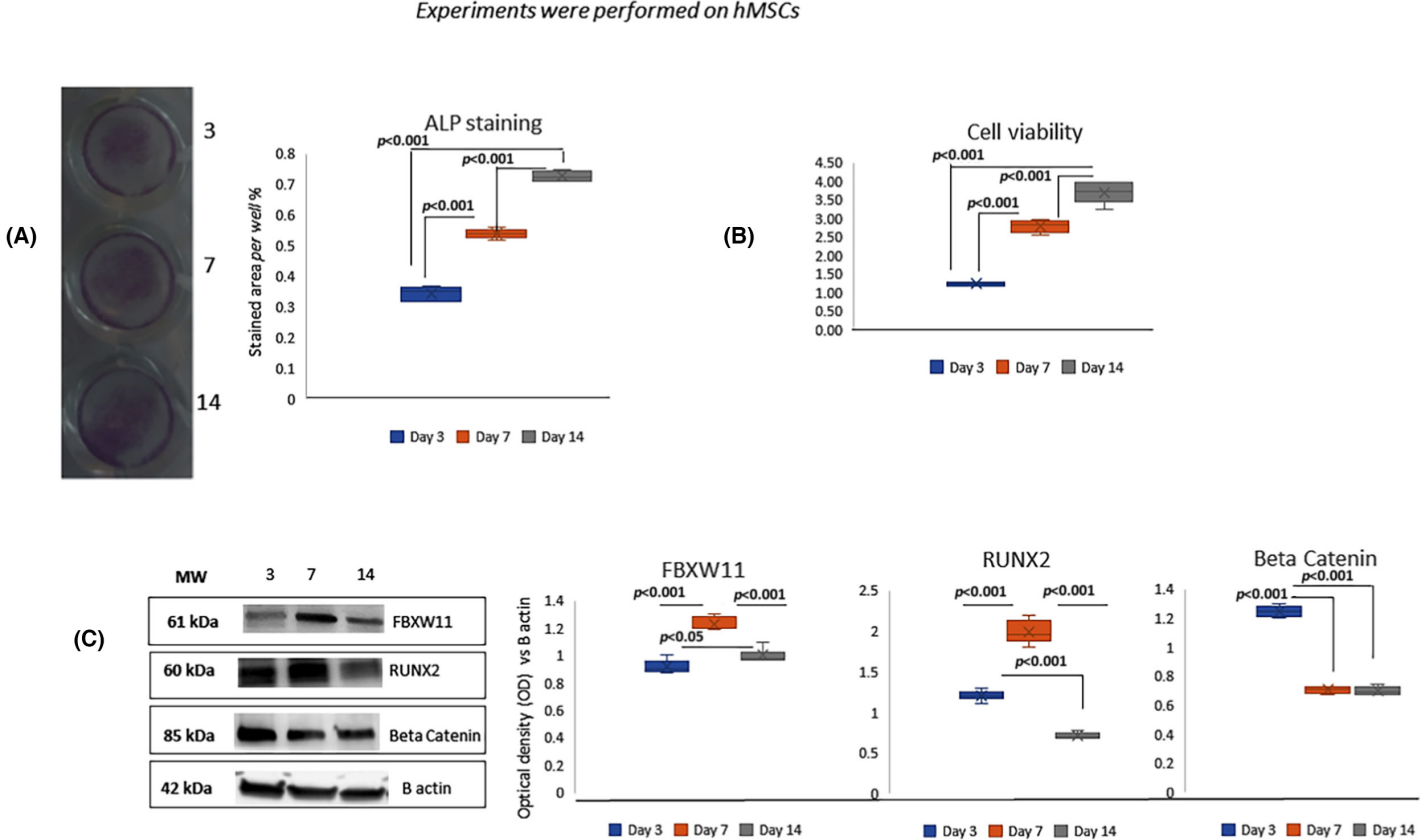
Osteogenic differentiation. ALP expression evaluation during osteogenic differentiation of hMSCs (A). hMSCs cells viability after 3, 7 and 14 days cultivation after osteogenic stimulation (B). WB for proteins levels (on the left) and optical density of the blots (on the right) (C). *p*‐Values were calculated by using one‐ way anova. For ALPL area staining and cell viability, six independent analyses have been performed. WB images are representative of three independent experiments and the graphs depict six densitometric analyses.

To evaluate the osteogenic differentiation, we performed ALP staining after 3, 7 and 14 days of differentiation. As shown in Figure 1A, the ALP staining area increased from 3 to 14 days of osteogenic stimulation. In particular, a stronger ALP staining was observed at 14 days of differentiation compared to 3 or 7 days of osteogenic differentiation. In addition, under the osteogenic stimulus the cells continued to grow throughout the observation period (1B). To understand the modulation of FBXW11 during osteogenic differentiation we evaluated its protein levels as well as those of the osteogenic master gene RUNX2 and beta‐catenin, involved in osteogenic commitment, after 3, 7 and 14 days of osteogenic differentiation. During the osteogenic differentiation, we observed increased protein levels of both RUNX2 and FBXW11 in the middle phase of differentiation (7 days of differentiation) (Figure 1C). After 14 days of differentiation, protein levels of FBXW11 started to decrease (Figure 1C). RUNX2 levels, on the other hand, dropped dramatically below those observed after 3 days of differentiation (Figure 1C). We also observed that beta‐catenin levels increased during the early osteogenic phase and lowered during osteogenic differentiation (Figure 1C).

### 3.2 FBXW11 regulates the expression of RUNX2and genes associated with osteogenic maturation

**FIGURE 2:**
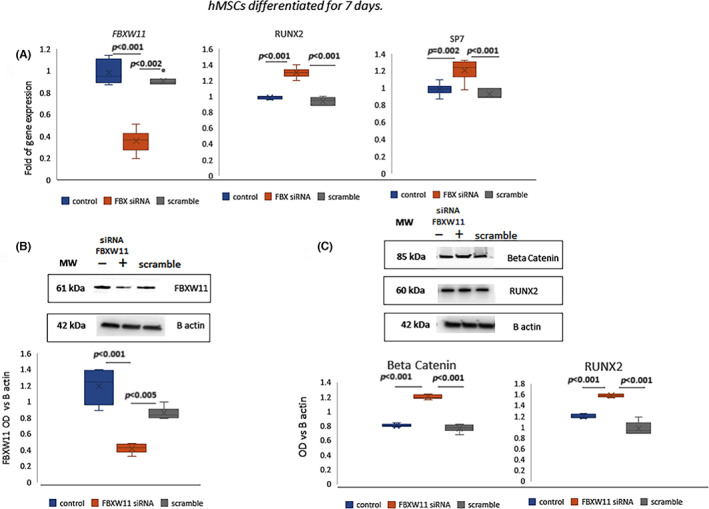
RUNX2, SP7 and beta‐catenin in FBXW11 silenced hMSCs. FBXW11, RUNX2, SP7 gene expression (A) and FBXW11 (B), beta‐catenin and RUNX2 (C) protein levels in controls and silenced hMSCs after 7 days of osteogenic differentiation. *p*‐Values were calculated by using one‐way anova. Gene expression has been evaluated in six independent experiments. WB images are representative of three independent experiments and the graphs show the data obtained by six densitometric analyses.

To evaluate the relationship between FBXW11 and RUNX2 in normal conditions, we silenced the expression of FBXW11 in MSCs cultured under osteogenic stimulation. In FBXW11‐silenced cells we also evaluated the expression of SP7, a RUNX2 downstream transcription factor essential for osteoblastogenesis. Furthermore, the association between SP7 and bone mineral density has been demonstrated in genome‐wide association studies.^28^ In FBXW11 silenced cells (Figure 2A,B) we observed an increased expression of RUNX2 and SP7 (Figure 2A). Furthermore, protein levels of RUNX2 as well as of its regulator beta‐catenin, increased in FBXW11 silenced cells (Figure 2C).

In order to evaluate the role of FBXW11 in osteogenic maturation, we analysed the expression of BGLAP (encoding bone gamma‐carboxyglutamate protein also known as osteocalcin), SPP1 (encoding osteopontin) and ALPL (encoding alkaline phosphatase) in FBXW11 silenced cells. In particular, ALPL is marker of osteogenic differentiation, BGLAP produces a protein secreted by osteoblasts and SPP1, in bone, is expressed by osteoblasts and osteoclasts.^29^

**FIGURE 3:**
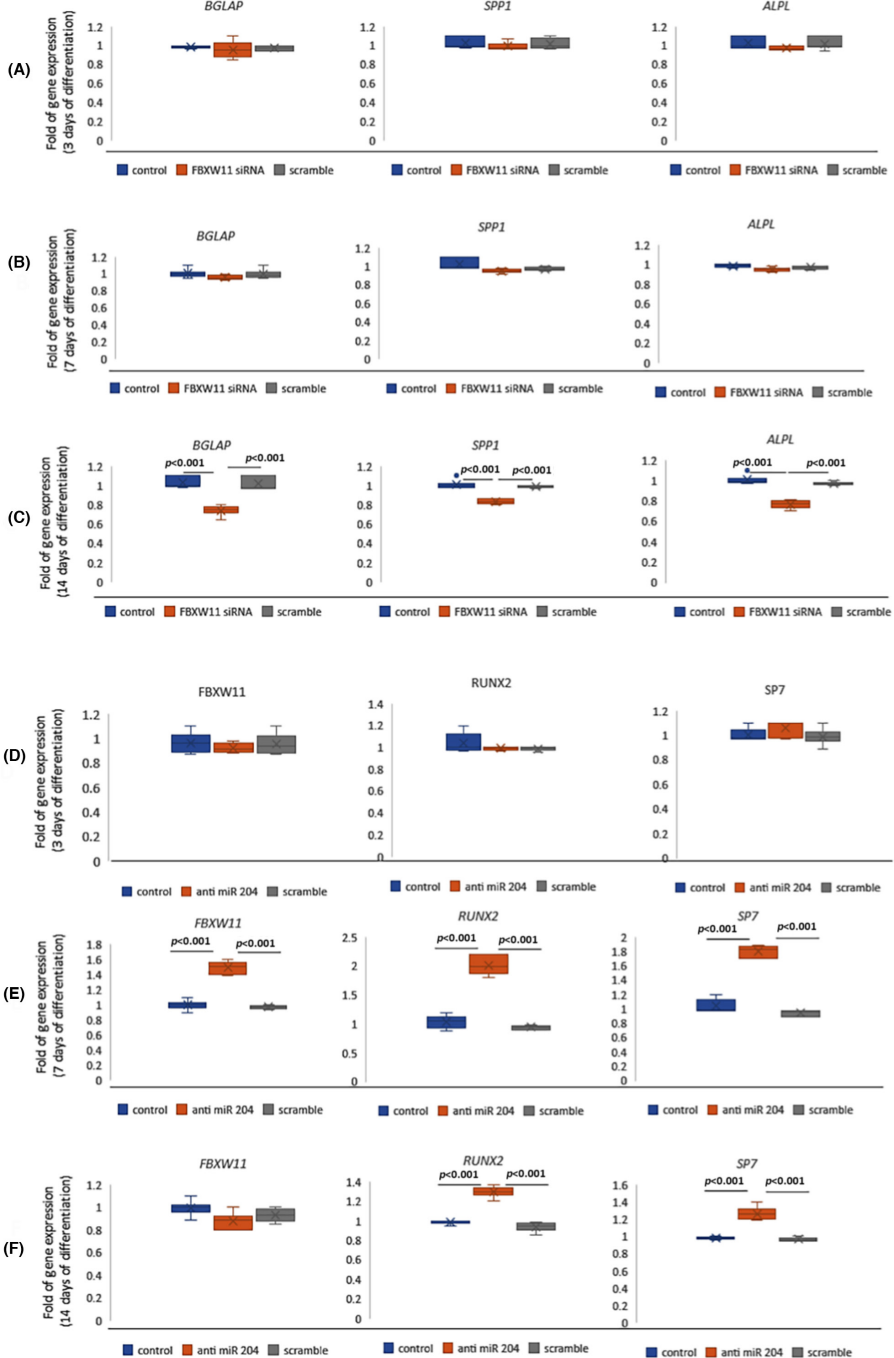
FBXW11 in osteogenic maturation. BGLAP, SPP1 and ALPL expression after 3 (A), 7 (B) and 14 days of differentiation in controls and FBXW11 silenced cells (C). FBXW11, RUNX2 and SP7 expression after 3 (D), 7 (E) and 14 (F) days of osteogenic differentiation in controls and RUNX2 forced expression‐hMSCs. Gene expression has been evaluated in six independent experiments. *p*‐Values were calculated by using one‐way anova.

The expression of genes associated with osteogenic maturation were similar in controls and FBXW11 silenced cells after 3 (Figure 3A) and 7 (Figure 3B) days of differentiation. However, we observed lower expression of BGLAP, SPP1 and ALPL after 14 days of differentiation in FBXW11 silenced cells compared to controls (Figure 3C).

We previously demonstrated that miR‐204 upregulates RUNX2 expression in the middle and late phase of osteogenic differentiation.^17^ Thus, by transfecting cells with anti‐miR 204 we did not observe FBXW11 and RUNX2 modulation after 3 days of osteogenic differentiation (Figure 3D). However, forced expression of RUNX2 after 7 and 14 days of osteogenic differentiation, obtained by transfecting MSCs with anti‐miR204, resulted in increased FBXW11 expression after 7 days (Figure 3E). However, this trend was not observed after 14 days of osteogenic differentiation (Figure 3F).

### 3.3 FBXW11 and RUNX2 show different modulation in CCD cells

**FIGURE 4:**
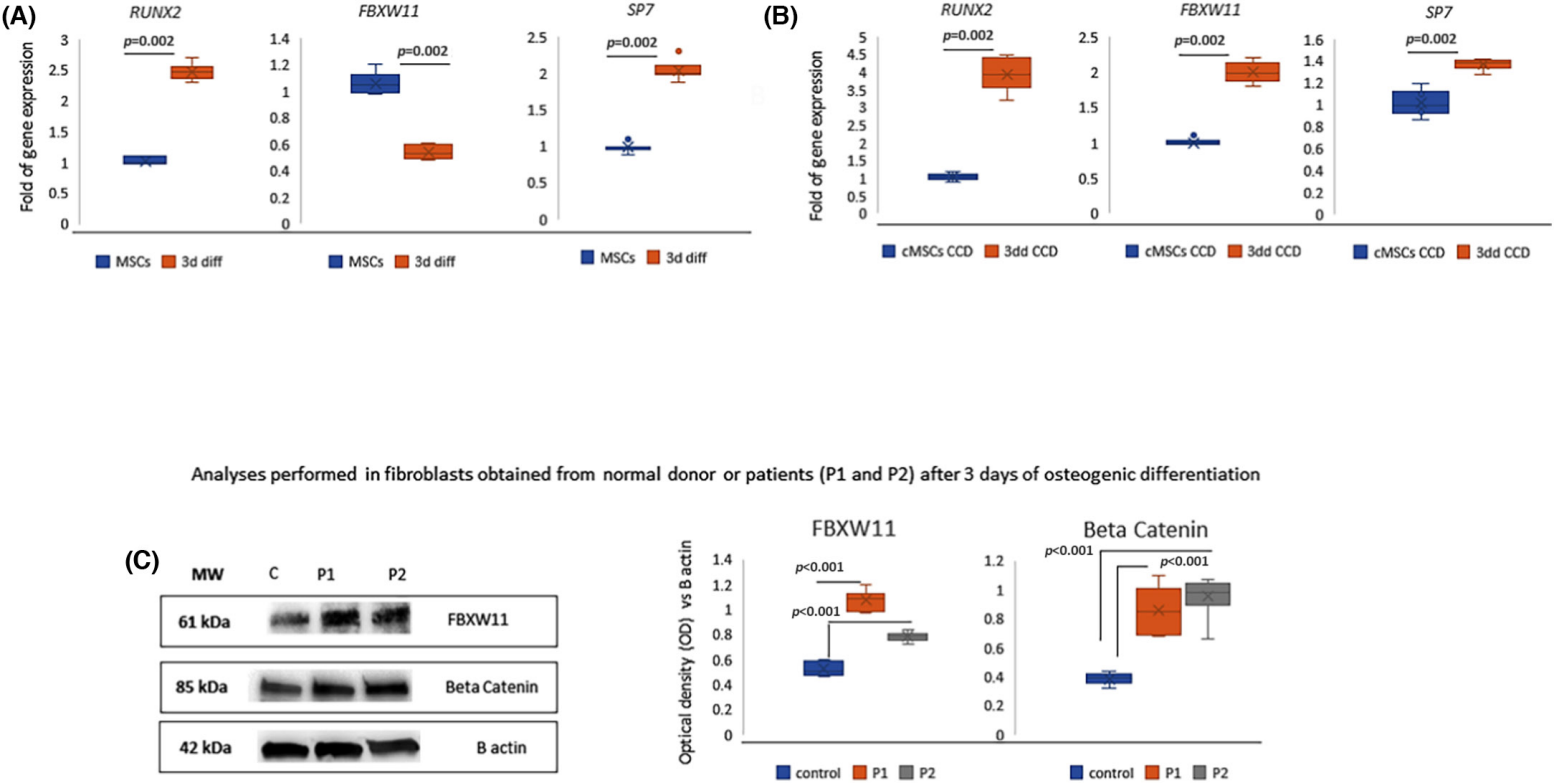
Gene expression or protein levels in CCD cells. RUNX2, FBXW11 and SP7 gene expression in undifferentiated MSCs line and after 3 days of osteogenic differentiation (A) and in CCD undifferentiated circulating MSCs and after 3 days of differentiation (B). Western blot in osteogenically stimulated fibroblasts obtained from normal donors or CCD patients (C). C, normal donors; P1, patient 1; P2, patient 2. *p*‐Values were calculated with the Mann–Whitney test (A and B) or one‐way anova (C). Gene expression has been evaluated in six independent experiments. WB images are representative of three independent experiments and the graphs show data obtained by six densitometric analyses.

To investigate the expression of FBXW11 during the osteogenic commitment, we evaluated FBXW11 expression in a MSCs cell line in the absence or presence of an osteogenic medium. Thus, RUNX2 and FBXW11 play an opposite modulation in MSCs and in the commitment of RUNX2 to osteogenic lineage. As shown in Figure 4A, RUNX2 expression, as well as the expression of the RUNX2‐dowstream transcription factor SP7, was higher while FBXW11 expression was lower during the commitment to osteogenic lineage (3 days of differentiation) than in MSCs.

To evaluate the modulation of FBXW11 in RUNX2 mutated cells, we first analysed the expression of FBXW11 in circulating MSCs (cMSCs) isolated from CCD paediatric patients and cultured in the absence or presence of osteogenic medium. These CCD‐cMSCS are CD105 and CD73 positive as previously reported.^23^

As shown in Figure 4B, FBXW11 levels increased associated with the increase of RUNX2 and SP7 levels during osteogenic commitment (after 3 days of osteogenic differentiation). To evaluate whether mutations at different sites of RUNX2 could modulate FBXW11 and beta‐catenin levels differently, we used patient fibroblasts with the c.897 T > G (Pt1) transition and patient fibroblasts with the c.1019del of a C nucleotide (Pt2). As shown in Figure 4C, the FBXW11 and beta‐catenin levels were higher in both osteogenically stimulated CCD fibroblasts compared to osteogenically stimulated fibroblasts of a normal donor (Figure 4C).

### 3.4 FBXW11 in osteosarcoma cells

**FIGURE 5:**
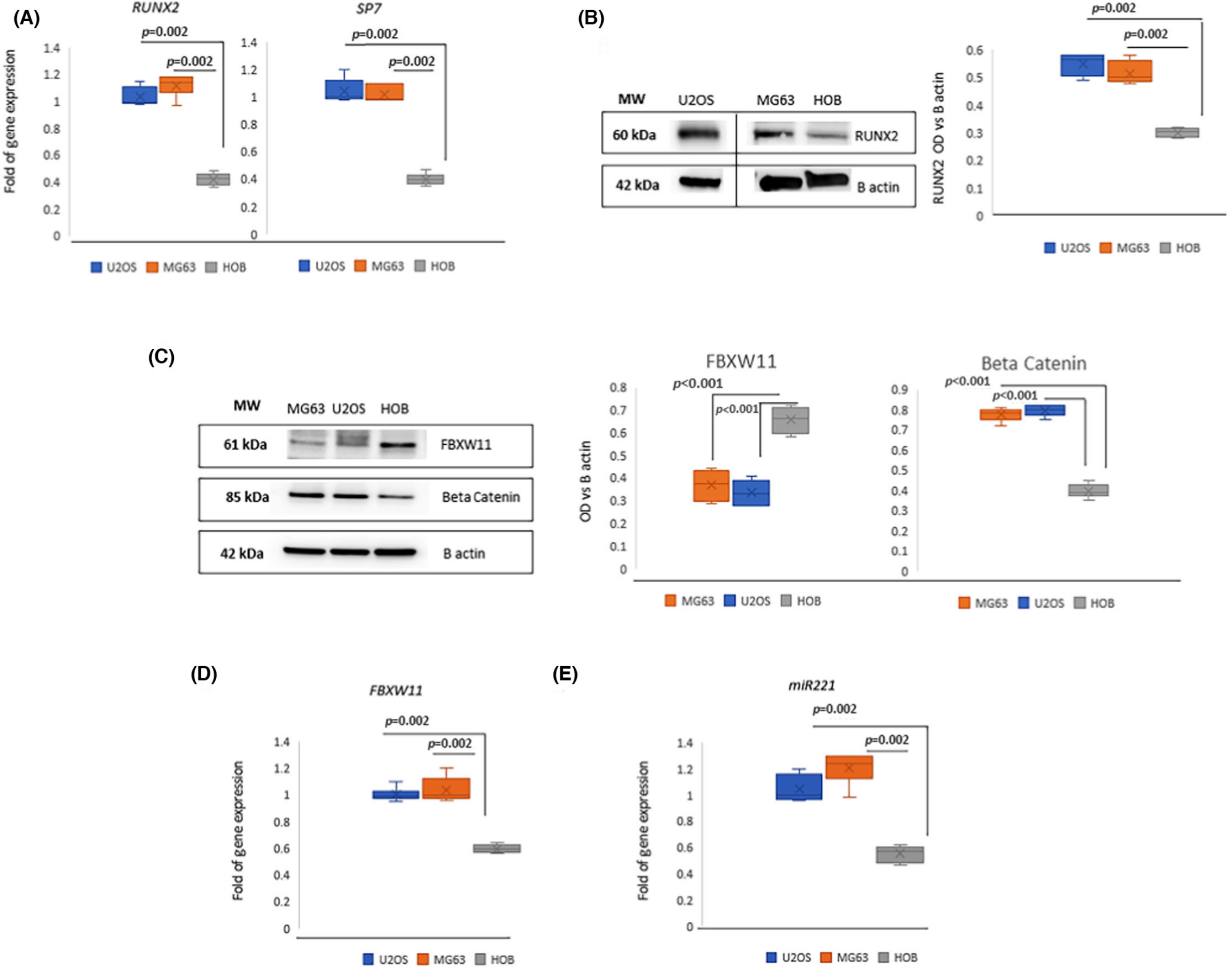
Analyses in osteosarcoma cells. RUNX2 and SP7 gene expression in U2OS and MG63 osteosarcoma cells and in normal osteoblasts (HOB) (A). RUNX2 protein levels in U2OS and MG63 osteosarcoma cells and in HOB; the dividing line indicates the spliced WB image (B). FBXW11 and beta‐catenin protein levels in MG63 and U2OS osteosarcoma cells and in HOB (C). Expression levels of FBXW11 (D) and of miR‐221 (E) in U2OS and MG63 osteosarcoma cells and in HOB. *p*‐Values were calculated with the Mann–Whitney test (A,B, D‐E) or one‐way anova (C). Gene expression has been evaluated in six independent experiments. WB images are representative of three independent experiments and the graphs show the data obtained by six densitometric analyses.

We analysed the expression levels of FBXW11 in normal osteoblasts and osteosarcoma cells expressing higher RUNX2 levels (Figure 5A,B). As shown in Figure 5C, we observed lower FBXW11 protein levels in MG63 and U2OS osteosarcoma cells compared to normal osteoblasts (HOB).

In addition, beta‐catenin levels were higher in osteosarcoma cells (MG63 and U2OS) compared to normal osteoblast (HOB) (Figure 5C). However, when we analysed FBXW11 gene expression levels in the U2OS and MG63 osteosarcoma cell lines we observed higher levels than in normal osteoblasts (Figure 5D). To understand the opposite trends of mRNA and protein levels of FBXW11 in osteosarcoma cells we analysed the expression of miR 221 which targets FBXW11.^30^ Indeed, we observed higher miR221values in the U2OS and MG63 osteosarcoma cell lines compared to normal osteoblasts (Figure 5E).

### 3.5 RUNT domain protects cells by proteasome degradation regardless of FBXW11 expression

**FIGURE 6:**
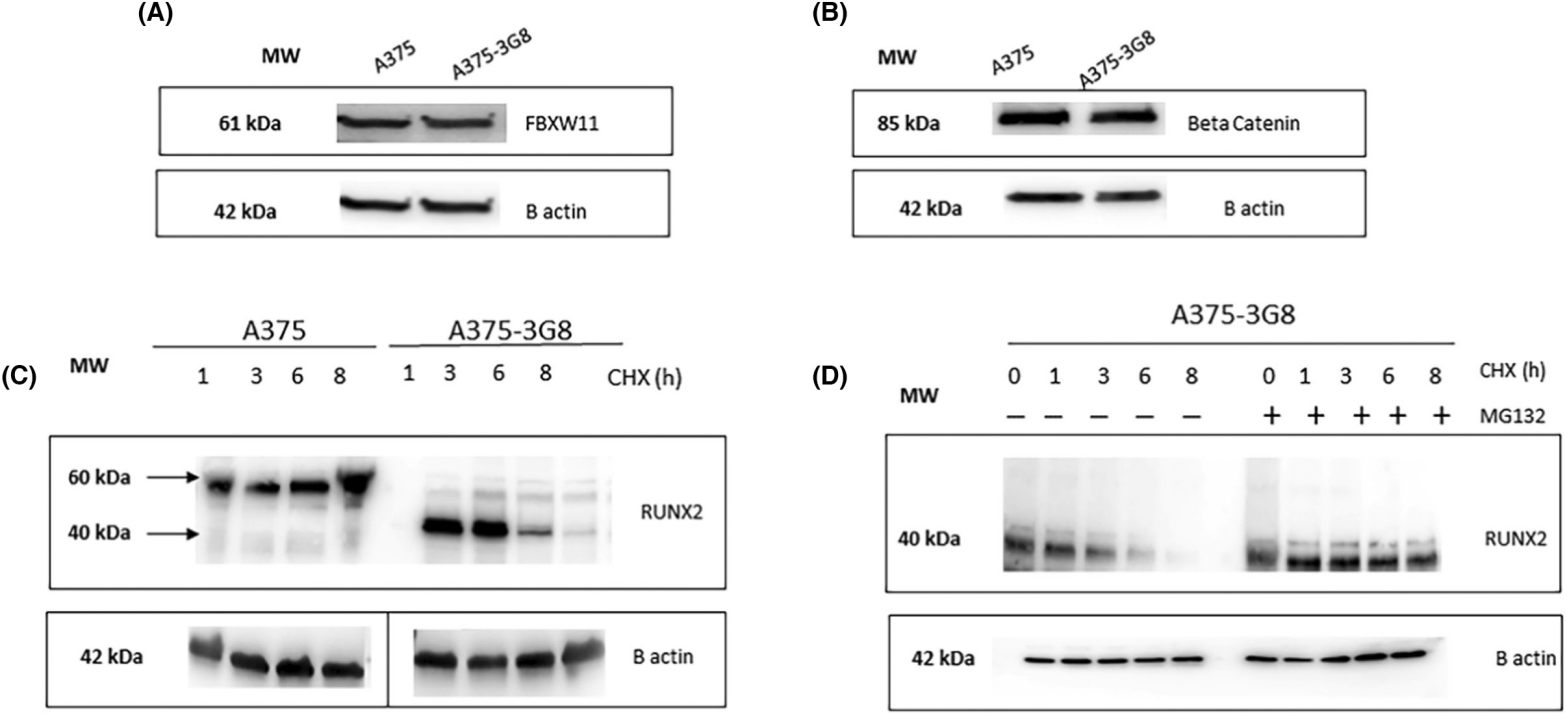
FBXW11 in parental or RUNT KO cells. FBXW11 (A) and beta‐catenin (B) protein levels in parental (A375) and RUNT KO (A375‐3G8) cells. RUNX2 protein levels in A375 and RUNT KO cells (A375‐3G8) treated with cycloheximide (CHX) from 1 to 6 h; the dividing line indicates the spliced WB image (C). RUNX2 protein levels in RUNT KO cells (A375‐3G8) treated without or with CHX and MG132 up to 8 h (D). (−: without MG132; +: in presence of MG132).

**SCHEME 1:**
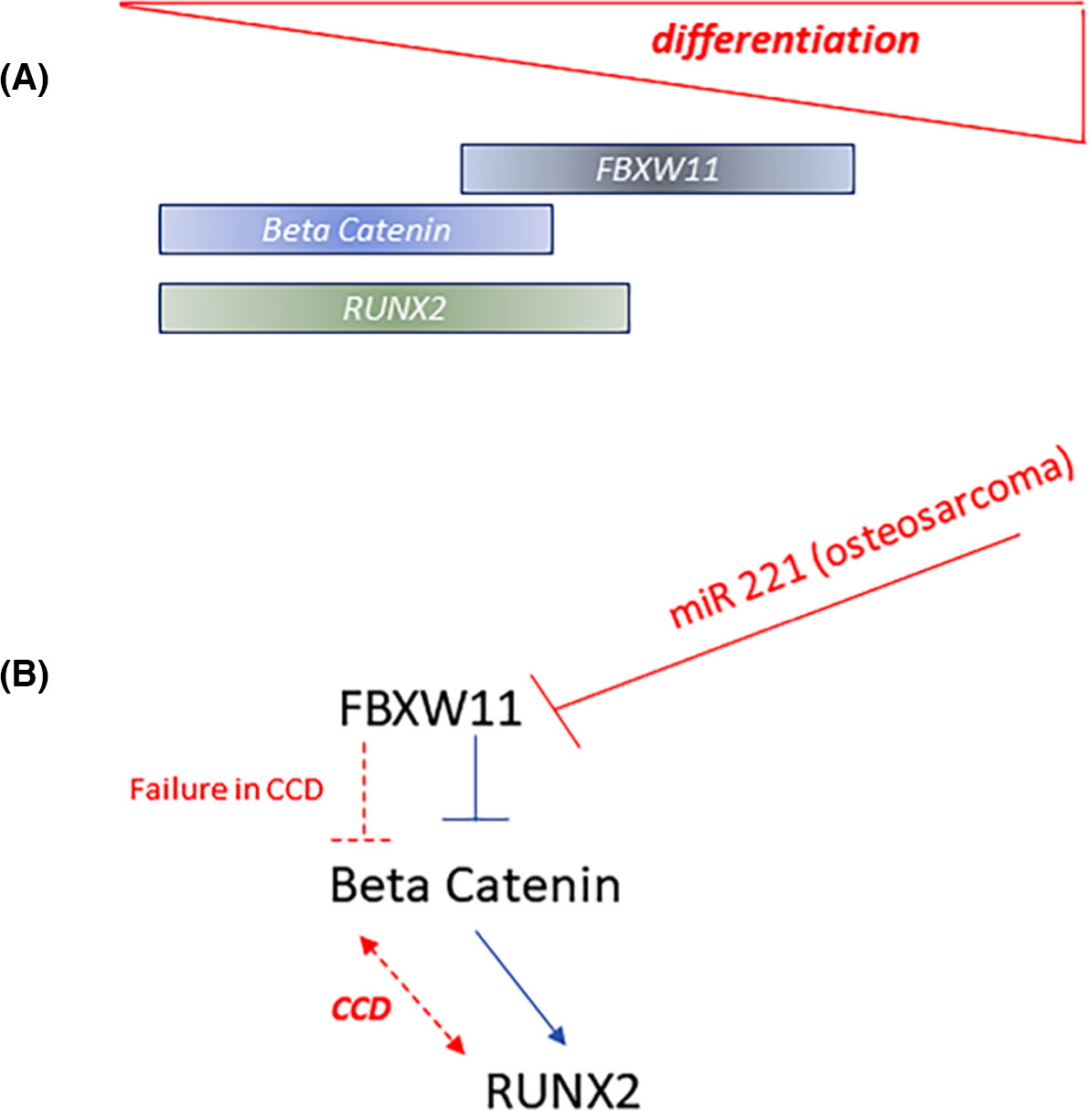
Schematic diagram of FBXW11 and osteogenesis. FBXW11 levels increase in the middle phase and beta‐catenin and RUNX2 levels increase in the early phase of osteogenesis (A). FBXW11, acting on the beta‐catenin target, reduces the levels of RUNX2 (blue lines B). In osteosarcoma, reduced FBXW11 protein levels, due to miR 221 overexpression, contribute to increase the levels of both beta‐catenin and RUNX2 (B). In the CCD model, mutated RUNX2 may contribute to the increase of beta‐catenin levels. In this disease model, FBXW11 is elevated in an attempt to counteract the dysregulated levels of beta‐catenin (red lines B).

To evaluate the role of FBXW11 in RUNX2 degradation by the ubiquitin‐proteasome system, we used the A375 line and A375‐derived cells in which RUNX2 RUNT domain had been deleted. In particular, we used these melanoma cells because, differing only in the RUNT domain, they represent an useful model to evaluate the role of RUNX2 in the degradation processes by the proteasome. Both cell lines expressed FBXW11 (Figure 6A) and beta‐catenin (Figure 6B) at similar levels. However, degradation of RUNX2, evaluated by inhibiting the proteome, was observed after 6 h only in cells with RUNT domain deletion (A375‐3G8) and not in the parental A375 cells (Figure 6C). The observed degradation in A375‐3G8 cells was prevented by inhibiting the proteasome with MG132 (Figure 6D), demonstrating the role played by RUNT domain, and not by FBXW11 expression, on proteasome‐driven degradation in this model (Scheme 1).

## 4 DISCUSSION

FBXW11 can modulate cell cycle and have stimulatory or inhibitory effects depending on its target proteins.^5^ In addition, it has been demonstrated that FBXW11 is involved in haematopoietic cells differentiation with specific modulation among long‐term or short‐term progenitor cells.^11^ It has also been reported that FBXW11 is involved in the proliferation process of cervical cancer,^31^ non‐small cell lung cancer,^10^ in lymphocytic leukaemia cells^12^ as well as in pancreatic cancer cells.^32^

However, even though the role of FBXW11 in cell cycle and differentiation as well as in tumorigenesis has been previously demonstrated, no studies have been performed to investigate the role of FBXW11 in osteogenesis. In this study, we observed increased expression levels of FBXW11 in the middle phase of osteogenesis. Importantly, during the middle and late phases of differentiation, we observed reduced expression of beta‐catenin. It has been reported that beta‐catenin is a target of FBXW11, and our results suggest a regulatory role of FBXW11 on RUNX2 through beta‐catenin modulation. These findings are supported by the data obtained upon FBXW11 silencing in osteogenic cells. In fact, we observed increased levels of both RUNX2 and beta‐catenin in FBXW11 silenced cells. However, in FBXW11 silenced cells we observed reduced expression of osteogenic maturation‐associated genes such as BGLAP, SPP1 and ALPL in the late phase of osteogenic differentiation. These data suggest that FBXW11 plays an important role for osteogenic maturation. Since it has been reported that RUNX2 expression has to be downregulated in the late phase of osteogenesis,^33^ the reduced levels of FBXW11, allowing continuous RUNX2 expression, may affect osteogenic maturation.

Furthermore, we also investigated the role of FBXW11 in forced expression of RUNX2, regardless of beta‐catenin activation. To this point, we inhibited the expression of miR‐204, a microRNA targeting RUNX2. Our results demonstrated an increase of FBXW11 expression after 7 days but not after 3 or 14 days of osteogenic differentiation. This finding suggests a role for FBXW11 during the middle phase of differentiation rather than during the early or late phase of osteogenesis. Accordingly, Wang et al.^11^ demonstrated that FBXW11 expression is higher in mature progenitors.

Several RUNX2 mutations have been associated with cleidocranial dysplasia (CCD, MIM #119600), a congenital disease affecting bone growth.^34^, ^35^

We previously identified novel C‐terminus RUNX2 mutations in CCD patients and observed RUNX2 overexpression in osteogenic differentiated cMSCs of these CCD patients compared to control cells.^23^ By culturing MSCs line in the presence or absence of osteogenic medium, we observed in this study an opposite modulation between FBXW11 and RUNX2. In particular, FBXW11 expression was higher in MSCs than in osteogenic committed cells. On the contrary, by performing gene expression analyses in circulating MSCs obtained from CCD patients and cultured in the presence or absence of osteogenic medium, we observed that both FBXW11 and RUNX2 levels increased during osteogenic commitment. In addition, FBXW11 levels were higher in osteogenically stimulated CCD fibroblasts compared to control. This last finding suggests that the overexpression of FBXW11 aims at adjusting mutated RUNX2 transcription factor levels. However, overexpression of FBXW11, and therefore the alteration of the ubiquitin‐proteasome system, could further contribute to impair osteogenesis in CCD patients. In fact, ubiquitin protease signalling is an important regulatory pathway in the differentiation process of bone marrow‐derived MSCs such as osteogenesis.^36^ Surprisingly, in osteogenically stimulated CCD Fibroblasts, we observed higher levels of beta‐catenin too. Since FBXW11 binds to its target proteins at phosphorylated sites to induce degradation,^12^, ^37^ the increased beta‐catenin in association with increased levels of FBXW11 suggests reduced phosphorylated beta‐catenin levels in CCD samples, emphasising the dysregulation of the cellular signal associated with RUNX2 mutations. In addition, we previously reported a reduced osteogenic maturation in fibroblasts of CCD patients compared to control.^23^ We were interested to evaluate FBXW11 expression also in the phase of osteogenic maturation. Unfortunately such evaluation was not feasible due to samples shortage and this represents a limitation of the work. Members of the UPS can be mutated or dysregulated in cancer.^38^ As reported in other malignancies, the ubiquitin‐proteasome system plays an important role in osteosarcoma too.^39^ Osteosarcoma is a consequence of impaired osteogenic differentiation due to genetic or epigenetic alterations.^40^ However, the FBXW11 expression modulation between osteosarcoma and normal osteoblast has been poorly investigated. Therefore, we analysed the FBXW11 expression in two osteosarcoma cell lines (MG63 and U2OS) and in primary human osteoblasts (HOB). Our data showed lower FBXW11 protein levels in MG63 and U2OS osteosarcoma cells compared to normal osteoblasts. We also observed that beta‐catenin levels were higher in osteosarcoma cells (MG63 and U2OS) compared to normal osteoblasts. However, by performing gene expression analyses we observed that FBXW11 was overexpressed in osteosarcoma cells compared to HOB. To explain these opposite findings among RNA and protein levels, we investigated the expression of miR‐221 which has been demonstrated to target FBXW11.^30^ Accordingly, miR‐221 was higher in osteosarcoma cells compared to HOB, suggesting that epigenetic alterations play an important role in osteosarcoma also by modulating FBXW11 expression.

Modulation of FBXW11 during osteogenesis or in bone cell‐related diseases suggests that FBXW11 affects cells via beta‐catenin and that FBXW11 and RUNX2 are closely associated. On the other hand, a direct effect of FBXW11 on the degradation of RUNX2 by the proteasome is unclear. To evaluate whether FBXW11 has a direct role in the degradation of RUNX2 by the proteasome system, we used a melanoma cellular model in which RUNX2 differs only in the RUNT domain. However, by using parental and RUNT KO melanoma cells, both expressing similar FBXW11 and beta‐catenin levels, we observed that RUNT domain is able to prevent proteasome degradation of RUNX2 regardless of the expression of FBXW11. These data suggest that FBXW11 is not sufficient to induce degradation of RUNX2 through the proteasome but other mechanisms are likely involved. Accordingly, it has been reported that Cbfb reduces the Cbfa‐1 ubiquitination‐mediated degradation.^41^ Cbfb is a co‐transcription factor forming a heterodimer with Runx2.^15^ In RUNT KO melanoma cells, lack of the RUNT domain may weaken Cbfb‐ RUNX2 binding and, consequently, expose it to degradation through the ubiquitin‐proteasome system.

Therefore, as schematized in the diagram, FBXW11, acting on beta‐catenin, indirectly reduces RUNX2 levels in osteosarcoma cells, whose FBXW11 levels decrease following miR 221 overexpression, increased levels of both beta‐catenin and RUNX2 are observed. Increased levels of RUNX2 caused by gene mutations such as in CCD induce an increase in beta‐catenin and, additionally, also in FBXW11 in response to increased beta‐catenin expression.

In summary, our data show that FBXW11 is highly modulated during differentiation and that increased levels of FBXW11 cause a decrease in the target beta‐catenin levels under normal conditions. Under pathological conditions such as CCD the relationship between FBXW11 and RUNX2 or beta‐catenin changes from normal conditions, suggesting the lack of beta‐catenin degradation by FBXW11. It has been reported that WNT signalling and RUNX2 mutually regulate their expression.^42^ In this disease model, FBXW11 levels might be elevated to counteract the dysregulated levels of beta‐catenin. Conversely, in osteosarcoma cells, the reduction of FBXW11 protein levels increases beta‐catenin. Since beta‐catenin promotes tumour formation and progression,^43^ reduced FBXW11 levels in osteosarcoma may be considered a negative prognostic factor. Our data therefore suggest that FBXW11 may be considered a prognostic factor in bone degeneration and that it may be included in a panel of bone tissue markers together with beta‐catenin and genes associated with osteogenic lineage commitment and maturation.

Therefore, all these results suggest that FBXW11 exerts its function specifically in the cellular context and, relatively to osteosarcoma, this protein can be considered an important marker for evaluating disease progression.

In conclusion, although further studies are needed to deepen the understanding of the observed molecular mechanisms, our study explored for the first time the expression of FBXW11 in normal and impaired osteogenic lineages and it confirms the involvement of UPS associated proteins in cell differentiation and alterations.

## AUTHOR CONTRIBUTIONS

**Luca Dalle Carbonare:** Conceptualization (equal); writing – original draft (equal). **Macarena Gomez Lira:** Supervision (equal). **Arianna Minoia:** Investigation (equal); methodology (equal). **Jessica Bertacco:** Investigation (equal); methodology (equal). **Silvia Orsi:** Investigation (equal). **Angela Lauriola:** Investigation (equal). **Veronica Li Vigni:** Investigation (equal). **Alberto Gandni:** Investigation (equal). **Franco Antoniazzi:** Supervision (equal). **Donato Zipeto:** Supervision (equal). **Monica Mottes:** Writing – review and editing (equal). **Lekhana Bhandary:** Validation (equal). **Daniele Guardavaccaro:** Writing – review and editing (equal). **Maria Teresa Valenti:** Conceptualization (lead); validation (lead); writing – original draft (lead); writing – review and editing (lead).

## FUNDING INFORMATION

This study was supported by Dalle Carbonare FUR (University of Verona).

## CONFLICT OF INTEREST STATEMENT

The authors declare no conflict of interest.

## INSTITUTIONAL REVIEW BOARD STATEMENT

The study was conducted according to the guidelines of the Declaration of Helsinki, and approved by the local ethical committee of Azienda Ospedaliera.

## INTEGRATA DI VERONA

Protocol code: 1538; 3 December 2012.

## INFORMED CONSENT STATEMENT

Informed consent was obtained from the parents of all subjects involved in the study. Children gave their assent for care.

## Data Availability

The data that support the findings of this study are available from the corresponding author upon reasonable request.
